# Editorial: From preconception to senescence: how do food and dietary interventions modulate health, immunity, and stress?

**DOI:** 10.3389/fnut.2024.1378632

**Published:** 2024-02-21

**Authors:** Jailane de Souza Aquino, Juliana Kelly da Silva Maia, Omar Guzmán-Quevedo

**Affiliations:** ^1^Department of Nutrition, Health Sciences Centre, Federal University of Paraíba, João Pessoa, PB, Brazil; ^2^Department of Nutrition, Health Sciences Centre, Federal University of Rio Grande do Norte, Natal, RN, Brazil; ^3^Tecnológico Nacional de México (TECNM)/Instituto Tecnológico Superior de Tacámbaro, Tacambaro, Michoacán, Mexico; ^4^Centro de Investigación Biomédica de Michoacán, Instituto Mexicano del Seguro Social, Morelia, Michoacán, Mexico

**Keywords:** phytochemicals, functional foods, nutraceuticals, diets, health properties, intermittent fasting, immunomodulatory, life cycle

Recent discussions pointed to the profound impact of food and dietary interventions on various facets of wellbeing, including immune function and stress management. The rising interest in different dietary patterns, diet interventions, like intermittent fasting, and the introduction of bioactive compounds in diets has continuously increased due to their potential for health maintenance, as well as prevention and treatment of diseases (1), in addition to immunomodulatory and anti-stress properties (2, 3).

These dietary interventions can contribute to health promotion from the preconception, the earliest life phases, until senescence. However, some dietary interventions can negatively affect different stages of life (1, 4). For this reason, this Research Topic aimed to publish articles that address the repercussions of dietary interventions including intermittent fasting, specific diets, and bioactive compounds on the modulation of health, immunity, and stress through the cycle of life. In this Research Topic, four articles were published, covering the aforementioned aspects.

Dietary restriction may imply weight loss and reduced systemic inflammation associated with obesity. An emerging strategy to decrease caloric intake is intermittent fasting. Regarding this, Mulas et al. conducted a review to assess how the two main forms of intermittent fasting (i.e., time-restricted eating and alternate-day fasting) impact body weight and key circulating inflammatory markers in adults with obesity. Results from this review indicate that time-restricted eating with various eating window durations (4–10 h per day) does not affect circulating levels of C-reactive protein (CRP), tumor necrosis factor-alpha (TNF-alpha), and interleukin-6 (IL-6); even with a weight loss of 1–5%. Regarding alternate-day fasting, reductions in CRP concentrations were observed when >6% weight loss was achieved. However, this did not affect TNF-alpha or IL-6 concentrations at this degree of weight loss. Thus, intermittent fasting has little or no effect on key inflammatory markers, but further research is needed to confirm these preliminary findings.

Senescence is characterized by a gradual and progressive deterioration of the immune system, due to the emergence of a pro-inflammatory state. Based on previous studies showing the ability of protein concentrates to modulate the immune response and cytokine levels, the paper by Miró et al. investigated whether diet supplementation with 8% spray-dried porcine plasma (SDP) improves the immunization capacity of senescent SAMP8 mice. Thus, 2-month-old mice were subjected to the diet supplemented for 4 months and challenged with 2.5 μg of *Staphylococcus aureus* enterotoxin B (SEB) at 4.5, 5, and 5.5 months of age. Then, a lethal shock was induced by intraperitoneal administration of SEB and lipopolysaccharide (LPS). All mice supplemented with SDP survived lethal shock vs. only 66% of control mice. SBE also reduced the expression levels of pro-inflammatory cytokines induced by lethal shock. This study shows the potential use of SDP to improve the effectiveness of vaccination in elderly people.

Gillies et al. conducted a clinical trial focusing on the rising popularity of flexitarian and exclusive plant-based diets among young adults. This surge in dietary preferences is influenced by factors such as health, cost-effectiveness, and increased environmental, and ethical awareness. This study explored the health, wellbeing, and behavioral implications of a basal vegetarian diet supplemented with red meat (flexitarian) or plant-based meat alternatives (PBMAs, vegetarian) over 10 weeks, involving 80 healthy young adults. Notably, the flexitarian group demonstrated higher adherence scores compared to the vegetarian group. Participants receiving red meat reported greater satisfaction, despite initial interest in plant-based eating. Both groups increased vegetable intake, reporting enhanced eating experiences and satisfaction. The study's success in encouraging trial engagement underscores implications for adopting sustainable dietary patterns in young adults, with the flexitarian group's consistent adherence suggesting potential for long-term adoption of healthy diets beyond the study. Therefore, emphasizing the need for robust measures in dietary intervention trials.

Nowadays, people are constantly exposed to stress factors impacting negatively on their health. Commonly, stress leads to sleep disturbances, subsequently affecting immunity. In this sense, Mohan et al. tested the effect of a proprietary black cumin oil extract (*Nigella sativa*) (BlaQmax^®^) on the stress-sleep-immunity axis. The double-blind study conducted in 72 people showed that a single daily dose of 200 mg/day for 90 days improves the functioning of the stress-sleep-immunity axis. In effect, the treatment reduced the level of stress as shown by reduced Perceived Stress Scale (PSS) scores on treatment days 45 and 90. Additionally, around 79% of participants reported satisfaction with their sleep patterns after 2 weeks of treatment. The volunteers also showed treatment-induced benefits on melatonin and cytokine levels. This study shows the suitability of the safe use of this dietary plant extract to combat the effects of stress on sleep and immunity disorders.

In summary, this Research Topic editorial emphasizes the ongoing and dynamic influence of food and dietary interventions, such as dietetic supplementation, intermittent fasting, and the adoption of flexitarian or exclusively plant-based diets on health, immunity, and stress across different life stages. Future research and public health initiatives should concentrate on formulating dietary recommendations that address the distinct needs of individuals at various points in life, thereby fostering a healthier and more resilient society.

## Author contributions

JA: Conceptualization, Investigation, Project administration, Supervision, Visualization, Writing—original draft, Writing—review & editing. JdS: Conceptualization, Investigation, Supervision, Writing—original draft, Writing—review & editing. OG-Q: Conceptualization, Investigation, Project administration, Supervision, Visualization, Writing—original draft, Writing—review & editing.
